# Evaluation of Different Copper Sources in Nile Tilapia Diets: Growth, Body Indices, Hematological Assay, Plasma Metabolites, Immune, Anti-Oxidative Ability, and Intestinal Morphometric Measurements

**DOI:** 10.1007/s12011-023-03570-x

**Published:** 2023-02-06

**Authors:** Mohamed A. EL-Erian, Mohamed S. Ibrahim, Shimaa M. R. Salem, Eman Y. Mohammady, Ehab R. El-Haroun, Mohamed S. Hassaan

**Affiliations:** 1 Central Laboratory for Aquaculture Research, Abbassa, Abou-Hammad, Sharkia, Egypt; 2 Department of Animal Nutrition and Nutritional Deficiency Diseases, Faculty of Veterinary Medicine, Mansoura University, Mansoura, Egypt; 3 Aquaculture Division, National Institute of Oceanography and Fisheries, NIOF, Cairo, Egypt; 4 Animal Production Department, Faculty of Agriculture, Cairo University, Cairo, Egypt; 5 Department of Animal Production, Fish Research Laboratory, Faculty of Agriculture at Moshtohor, Benha University, Banha, 13736 Egypt

**Keywords:** Bulk-Cu, Nano-Cu, *Oreochromis niloticus*

## Abstract

The goal of the current study was to compare how well Nile tilapia, *Oreochromis niloticus*, utilized copper (Cu) as bulk and nano sources by evaluating fish growth, body indices, hematological assays, plasma metabolites, immune and anti-oxidative abilities, and intestinal morphometric measurements. The basal diet served as a control, with no Cu added, whereas the experimental diets were formed by adding bulk-Cu and nano-Cu to the basal diet to keep Cu levels at 3 and 6 mg kg^−1^, respectively, in both Cu sources. Tilapia (9.10 ± 0.014 g) were fed the control diet and four experimental diets for 12 weeks. Results indicated that growth, feed utilization, and body indices demonstrated a substantial improvement (*P* ≤ 0.05) in tilapia that received a diet containing 3 and 6 Nano-Cu mg kg^−1^ diet, compared to the performance of fish that received diets containing 3 and 6 Bulk-Cu mg kg^−1^ diet and the control diet. Villi height, villi width, absorption area of villous (AAV), and mucosal to serosal amplification ratio (MSR) values demonstrated a substantial increase (*P* ≤ 0.05) in tilapia fed 3 and 6 mg kg^−1^ Nano-Cu compared to the values observed in fish fed the control and Bulk-Cu supplemented diets. Fish fed Bulk-Cu recorded the highest (*P* ≤ 0.05) hemoglobin concentration in those fed 6 mg kg^−1^ compared to 3 mg kg^−1^. Hematocrit value considerably improved (*P* ≤ 0.05) by supplementation of Cu, whereas the highest significant value demonstrated in fish provided 6 mg/kg^−1^ Nano-Cu. A fish-fed diet containing 3 mg kg^−1^ Nano-Cu revealed the best (*P* ≤ 0.05) values of plasma albumin, total protein, and globulins. Plasma HDL-C highest concentrations (*P* ≤ 0.05) were reported in fish fed diet supplemented with 6 mg/kg^−1^ either Bulk or Nano Cu, whereas values of plasma TG and VLDL-C declined as Cu supplementation level increased either from Bulk or Nano source. Also, the best (*P* ≤ 0.05) values of CAT and GPX were seen in fish given diet supplemented with 6 Nano-Cu mg/kg^−1^. Fillets of fish-fed Nano-Cu-supplemented diets showed a marked decline (*P* ≤ 0.05) in moisture and fat contents, while crude protein, ash, and Cu contents considerably increased in the fillet by dietary supplementation of Nano-Cu at both levels 3 and 6 mg kg^−1^. In conclusion, the supplemental diets with 3 or 6 Nano-Cu mg/kg^−1^ enhanced growth, feed utilization, body indices, fillet nutrient composition, hematological assay, plasma metabolites, immune, antioxidant activities, and intestinal morphometry of Nile tilapia.

## Introduction

Nile tilapia (*Oreochromis niloticus*) is now one of the most widely cultivated tropical fish worldwide that has become a highly profitable species in aquaculture, with a global production of 5.5 million MT in 2018 [1]. Minerals are needed to maintain an animal’s regular physiological and metabolic processes. A deficiency of certain required minerals in the diet can result in a variety of health problems.

All organisms, including fish, require copper (Cu), a dietary essential trace element. It serves as a cofactor of particular proteins and enzymes, including tyrosinase, ferroxidase, superoxide dismutase, cytochrome oxidase, and dopamine hydroxylase [2]. These enzymes stimulate different metabolic processes, including antioxidant reactions by scavenging free radicals, also involved in electron transport, and the formation of hemoglobin and collagen [3].

Copper is obtained by fish through the water and their diet. However, copper present in water is insufficient to meet the requirements, and dietary sources are essential for Cu procuration in aquatic animals. Waterborne copper only represents about 10% of whole-body copper content, while a diet containing 0.8 mg/kg copper increases the copper content up to 60% [4]. Typical feedstuffs used in fish feed formulation, such as fishmeal and plant protein sources, contain insufficient Cu to cover the fish’s needs [5–7]. Thus, it becomes necessary to supplement Cu to formulated fish feed to adjust the Cu content to the proper level required by fish.

Adverse effects of Cu deficiency have been observed in many fish species, such as reduced growth of grouper, lower efficiency of cytochrome C oxidase in the heart, copper‑zinc superoxide dismutase in the liver of channel catfish *Ictalurus punctatus*, alkaline phosphatase, and acid phosphatase in blunt snout bream [7, 8]. On the other hand, excess Cu intake has a toxic effect and can delay growth in rainbow trout and tilapia *Oreochromis niloticus* × *O. aureus* [9, 10]. This toxic effect may relate to the deterioration of body cells and organs, as the extra copper ion stimulates the synthesis of reactive oxygen species that destroy lipids, proteins, and DNA [11, 12].

Dietary copper requirements have been established in different fish species, such as rainbow trout, Atlantic salmon, common carp, channel catfish, grass shrimp, tilapia, grouper, yellow catfish, grass carp, blunt snout bream, large yellow croaker, tongue sole, red sea bream, Russian sturgeon and abalone [7, 8, 10, 13–24]. The dietary requirements of copper depend on the fish species, age, and feeding plan [14, 25]. Shiau and Ning [10] have detected the copper requirement for Tilapia *Oreochromis* at 5 mg kg^−1^ DM.

Well, understanding Cu bioavailability from Cu supplements is an important factor that should be considered during the selection of a Cu source [26]. High bioavailability of Cu in fish diet can decrease the need for Cu supplements and the excreted Cu in wastes, which accumulate, pollute the rearing water, and harm the fish’s health. Inorganic Cu salts are commonly used as feed supplements for growth promotion, such as Cu sulfate, Cu oxide, and Cu chloride.

Nowadays, nanoparticles of metals are involved in many sectors as agriculture, industry, aquafeed. It can be used as a growth promoter, antioxidant, and immunostimulant in fish [23, 43]. Nano or chelated form of Cu showed better bioavailability compared to the inorganic form in fish, such as rainbow trout, grouper, and channel catfish [27, 28]. Available data about the contribution of nano Cu molecules as a dietary supplement are limited; available findings from previous investigations revealed that Cu nanoparticles have improved biological effects and availability compared to inorganic Cu salts in piglets and fish, such as red sea bream [23]. It may be due to the small size of nano Cu facilitating and increasing its absorption via endocytosis and cell bypass mechanisms [29]. Thus, the present study was designed to investigate the bioavailability of Bulk-Cu and Nano-Cu for satisfying the dietary Cu requirement for Nile tilapia, by evaluation of fish growth traits, whole-body copper status, blood hematology, antioxidant activities, immune responses, intestinal morphology, and serum biochemical parameters.

## Material and Methods

### Experimental Diets Preparation

The control diet (Diet 1) (Table 1) was designed following Nile tilapia’s requirements recommended by [14]: 32% crude protein and 6% lipid. Cu was supplemented from Bulk (CuSO_4_) and Nano sources. Nano-copper (Sigma-Aldrich, 207,780-500G: 99%, USA) was used as the Cu source. The sizes of the elemental nano-Cu particles < 75 μm. The Bulk-Cu was added to the control diet at 3 mg kg^−1^ and 6 mg kg^−1^. Also, Nano-Cu was added to the control diet at 3 mg kg^−1^ and 6 mg kg^−1^ diet. Diets were well combined with Bulk-Cu and Nano-Cu before 300 ml of water kg^−1^ was added to create a dough. The feed mixture was pelletized using a lab pelletizer with a 2-mm-diameter die. The feed pellets were dried for 24 h at ambient temperature before being stored until usage in a 4 °C refrigerator. The proximate composition of the nutrients in the experimental diets was examined using the methods described in [30] (Table 1). By utilizing an atomic absorption spectrophotometer, the dietary Cu contents were calculated as follows: 13.62 (control), 16.52 (Diet 2), 19.32 (Diet 3), 16.70 (Diet 4), and 19.12 (Diet 5) mg kg^−1^ diet.

**Table 1:** Ingredients and proximate nutrient composition percentage of the control diet (g/kg diet, dry matter)

Ingredients	%
Fish meal (65%)	10
Soybean meal (45%)	46.3
Corn gluten meal	3.00
Yellow corn	16.6
Wheat flour	18.5
Soybean oil	3.4
Vitamin mixture^*^	0.8
Mineral mixture^**^	0.5
DiCaP	0.6
Choline chloride	0.2
Stay C	0.1
Proximate analysis (%)	
Crude protein	32.1
DE (Kcal/kg)	3001
Crude lipid	5.74
Ash	5.169
Crude fiber	4.11
Ca	0.798
P	0.825
Cu (mg/kg)	13.700

^*^Vitamin mixture (g/kg premix): Thiamin HCl, 0.44; Riboflavin, 0.63; Pyridoxine HCl, 0.91; DL pantothenic acid, 1.72; Nicotinic acid, 4.58; Biotin, 0.21; Folic acid, 0.55; Inositol, 21.05; Menadione sodium bisulfite, 0.89; Vitamin A acetate, 0.68; Vitamin D3, 0.12; dL-alpha-tocopherol acetate, 12.63; Alpha-cellulose, 955.59

^**^Mineral mixture, copper-free (g/100 g premix): Cobalt chloride, 0.004; Ferrous sulfate, 4.000; Magnesium sulfate anhydrous, 13.862; Manganous sulfate monohydrate, 0.650; Potassium iodide, 0.067; Sodium selenite, 0.010; Zinc sulfate heptahydrate, 13.193; Alpha-cellulose, 67.964

Abbreviations: *Stay C®* (L-ascorbyl-2-polyphosphate 35%, *DE* digestible energy, *Ca* calcium, *P* phosphorus, Cu copper

### Experimental Procedure

A private farm in Kafer Elsheikh provided Mono-sex Nile tilapia fingerlings to use in this trial. Fish were fed the control diet throughout the 2-week adaption period in two circular fiberglass tanks (1 m^3^) in the laboratory of fish in Abbassa, Abou-Hammad, Sharkia Governorate, Egypt, before the experiment started. After adaptation, the fish fasted for 24 h. Healthy Nile tilapia (9.10 ± 0.014 g) were randomly allocated to 15 tanks (80 × 50 × 50 cm; 200 L for each), representing the five groups with three replicates, 30 fish per aquarium. Fish were fed the test diet by hand at 09:00, 12:00, and 15:00 for a period of 12 weeks in three equal meals. Fish were fed 3% of their body weight each day. Every 2 weeks, fish were weighed, and the daily ratio was changed in accordance with weight gain. The experiment was conducted with water that was maintained at a temperature of 26.9 ± 0.50 °C, dissolved oxygen levels of 5.65 ± 0.21, a pH of 8.30 ± 0.30, and total ammonia levels of 0.19 ± 0.01 mg l^−1^. During the study period, a photoperiod with 12 h of light (08:00 to 20:00 h) and 12 h of darkness was used.

### Growth and Body Indices Estimation

The growth performance of the juvenile Nile tilapia was calculated using weight gain (WG), feed conversion ratio (FCR), the protein efficiency ratio (PER), specific growth rate (SGR), survival %, condition factor (CF), calculated as equations noted by [30].

### Sample Collection

At the conclusion of the growth period, fish were deprived of feed for 24 h and then anesthetized with tricaine methanesulfonate (MS222) at 150 mg l^−1^. To estimate the final body weight, weight increase, and survival rates, the total number and weight of fish in each tank were recorded. Blood samples were taken from the caudal vein of three fish per group by using 10% ethylenediaminetetraacetate (EDTA), then separated into two groups. The first blood group was separated to test the parameters of hematology, while the blood from the second group was centrifuged for 10 min at 3000 g to obtain the blood plasma. The obtained plasma samples were saved at − 20 °C for further analysis. After blood collection, individual fish weight and length were recorded for later estimation of the condition factor. Then, fish were dissected, and samples from the anterior and posterior intestines were separated for histomorphometry determination. Intestinal samples were fixed in 10% neutral-buffered formalin until examination [31]. Additionally, the other three fish from each treatment were anesthetized by MS222 at 150 mg l^−1^, homogenized, dried, and stored at − 20 °C for subsequent fish flesh proximate and Cu content analysis.

### Sample Analyses

#### Blood assay

Hematocrit was analyzed according to [32] procedures. The indirect approach described by [33] was used to count the RBCs and WBCs, and hemoglobin (Hb) was quantified using hemoglobin kits (cat. no. KT-731), which is a standardized procedure of the cyanmethemoglobin method. The methods described by [34] was used to quantify the mean corpuscular volume (MCV), mean corpuscular hemoglobin (MCH), and mean corpuscular hemoglobin concentration (MCHC). By multiplying the Hb content by 1.25 oxygen and adding the power of the Hb g^−1^, the oxygen carrying capacity was determined [35].

Following instructions, the blood plasma’s total protein and albumin were each examined [36, 37], respectively. The calculation of globulin, however, was done by subtracting albumin from total protein, as stated in [36]. Plasma levels of the three enzymes aspartate aminotransferase (AST), alanine aminotransferase (ALT), and alkaline phosphatase were measured using the method described by [32, 37], and plasma creatinine was quantified using the colorimetric and enzymatic determine cation methods as shown by [37]. Estimations of plasma total cholesterol, triglyceride, high-density lipoprotein cholesterol (HDL-C), and low-density lipoprotein cholesterol (LDL-C) were used [38].

#### Plasma Immune and Antioxidant Biomarkers

According to [39], plasma lysozyme activity was assessed using Micrococcus lysodeikticus as a model. Based on the approach described in [33], the plasma activities of superoxide dismutase (SOD), catalase (CAT), glutathione peroxidase (GPx), and malondialdehyde (MDA) were determined.

### Histomorphometry Examination of the Intestine

Using a Rotatory Microtome (Reichert Technologies), the intestine longitudinal and transverse slices, each 6 m thick, were cut and stained with hematoxylin and eosin by usual protocol. [40]. The light microscope was supplied with a microscopic camera, and image processing software Olympus LC20 was fixed on an Olympus microscope (Olympus BX-50) with a 1/2 × image adapter, and a 40 × objective was used to examine the tissue sections. Image analysis software was used to calculate the mean villus height (measured from the base to the top) for statistical analysis. The area of the absorption surface was determined as described by [41].

### Fish Fillet Proximate Analysis

The proximate chemical analysis of fish fillets was analyzed following the technique revealed by [42]. After the samples were dried in an oven (105 °C) for 24 h, dry matter was measured. Using a Kjeltechauto analyzer, Model 1030 from Tecator, crude protein was evaluated using the micro-Kjeldahl method, N% 6.25, and crude fat was determined using the Soxhlet extraction method with diethyl ether (40–60 °C). Using ignition at 550 °C for 12 h, ash was identified. An atomic emission spectrophotometer (IRIS Advantage; Thermo Jarrell Ash Corporation) was used to measure the amount of copper in fish fillets using standard copper concentrations [42].

### Statistical Examination

Using SAS version 9.4 software, all data were examined (SAS, 2016). To identify the significant difference across various treatments, one-way analysis of variance (one-way ANOVA) and the [44] new multiple-range tests were applied. The data are shown as means with pooled standard error of the mean since all differences were found significant at *P* < 0.05.

## Results

### Growth Indices

Table 2 displays the growth, feed utilization, and body indices data. Growth, feed utilization, and body indices demonstrated a substantial improvement (*P* ≤ 0.05) in tilapia that received a diet containing 3 and 6 Nano-Cu mg kg^−1^ diet, compared to the performance of fish that received diets containing 3 and 6 Bulk-Cu mg kg^−1^ diet and the control diet. Additionally, the tilapia survival rate increased considerably (*P* ≤ 0.05) by dietary supplementation of Cu either from Bulk-Cu or Nano-Cu.

**Table 2:** Growth performance, feed utilization, and biological parameters of Nile tilapia as affected by dietary additives of bulk and Nano copper

	Experimental treatments					± MSE	*P* value
	Control	Bulk-Cu (mg kg^−1^ diet)		Nano-Cu (mg kg^−1^ diet)			
		3	6	3	6		
IBW (g fish^−1^)	9.09	9.08	9.10	9.11	9.12	0.014	0.2903
FBW (g fish^−1^)	41.14^c^	48.18^b^	47.47^b^	52.67^a^	52.10^a^	1.02	0.0001
FBL	13.03^d^	14.30^b^	14.10^c^	14.77^a^	14.70^a^	0.56	0.0001
K	1.86^a^	1.65^b^	1.69^b^	1.64^b^	1.64^b^	0.53	0.0003
WG (g fish^−1^)	32.05^c^	39.10^b^	38.37^b^	43.56^a^	42.98^a^	1.11	0.0001
SGR (% day^−1^)	1.79^c^	2.00^ab^	1.99^b^	2.05^a^	2.05^a^	0.02	0.0001
FS (%)	80.00^b^	93.33^a^	96.67^a^	100^a^	100^a^	1.17	0.0017
FI (g fish^−1^)	59.61^d^	68.42^b^	65.24^c^	71.44^a^	70.77^ab^	1.41	0.0001
FCR	1.86^a^	1.75^b^	1.70^c^	1.65^d^	1.64^d^	0.54	0.0001
PER	1.79^d^	1.90^c^	1.96^b^	2.03^a^	2.02^a^	0.21	0.0001

Means followed by different small letters in the same row are significantly different (*P* < 0.05, one-way ANOVA)

Abbreviations: *IBW* initial body weight, *FBW* final body weight, *FBL* final body length,* K* condition factor, *WG* weight gain, *SGR* specific growth rate, *FS* fish survival, *FI* feed intake, *FCR* feed conversion ratio, *PER* protein efficiency ratio

### *Intestinal Histopathological Measurements*

The dimensions of the anterior and posterior intestine components are listed in Table 3. The villi height showed the significantly highest values by dietary supplementation of Nano-Cu at both levels 3 and 6 mg/kg^−1^. Villi width, goblet cells number, and mucosal to serosal amplification ratio (MSR) values demonstrated a substantial increase (*P* ≤ 0.05) in tilapia fed 6 mg kg^−1^ Nano-Cu compared to the values observed in fish fed the control and Bulk-Cu supplemented diets, while the best absorption area of villous (AAV) value (*P* ≤ 0.05) was noticed in fish receiving 3 mg/kg^−1^ Nano-Cu. Further, crypt width values decreased significantly (*P* ≤ 0.05) in fish given 6 mg kg^−1^ Nano-Cu and the control diet, in contrast to the values in the other treatments. Similarly, dimensions of the posterior intestine components improved (*P* ≤ 0.05) by dietary supplementation of 3 and 6 mg/kg^−1^ Nano-Cu, except, the villi width value which was the highest (*P* ≤ 0.05) in fish fed 6 mg/kg^−1^ Nano-Cu. Also, the goblet cell number was not significantly altered (*P* ≥ 0.05) in fish fed different dietary treatments. Finally, the value of crypt width revealed the dramatic (*P* ≤ 0.05) highest value in fish-fed diets supplemented with 6 mg kg^−1^ Bulk-Cu.

**Table 3:** Histomorphometric *of intestine of Nile tilapia as affected by dietary additives of* bulk and Nano Copper

	Experimental treatments						± MSE		*P* value
	Control	Bulk-Cu (mg kg^−1^ diet)		Nano- Cu (mg kg^−1^ diet)					
		3	6	3	6				
*Anterior intestine*									
Villi height	322.15^c^	479.52^b^	489.33^b^	685.81^a^	691.52^a^		3.69		0.001
Villi width	30.25^c^	33.87^b^	334.08^b^	32.52^b^	38.62^a^		0.562		0.001
Goblet cells number	27.12^c^	33.00^c^	39.90^b^	40.19^ab^	47.42^a^		0.236		0.001
MSR	8.27^c^	8.82^c^	8.35^c^	13.29^b^	16.17^a^		0.452		0.001
AAV	45.66^b^	46.13^b^	44.46^b^	66.29^a^	47.95^b^		0.598		0.001
Crypt width	58.42^b^	62.13^a^	66.02^a^	62.51^a^	48.92^c^		1.89		0.002
*Posterior intestine*									
Villi height	186.22^d^	192.58^c^	268.59^b^	274.33^a^	292.69^a^	20.23		0.039	
Villi width	52.23^c^	66.22^b^	75.89^b^	78.51^b^	89.26^a^	5.69		0.008	
Goblet cells number	43.58	42.00	41.09	41.01	42.05	0.987		0.154	
MSR	4.29^a^	5.82^b^	6.12^b^	9.05^a^	9.57^a^	0.878		0.001	
AAV	10.65^c^	12.08^c^	13.04^c^	15.30^a^	18.20^a^	2.56		0.702	
Crypt width	42.03^b^	48.65^b^	59.12^a^	38.93^c^	35.65^c^	1.59		0.001	

Means followed by different letters in the same row are significantly different (*P* < 0.05, one-way ANOVA)

Abbreviations: *MSR* mucosal to serosal amplification ratio, *AAV* absorption area of villous

### Blood Assay

Blood assay measurements are summarized in Table 4. Hemoglobin concentration was improved (*P* ≤ 0.05) by dietary supplementation of Cu either from Bulk or Nano-Cu, whereas fish fed Bulk-Cu recorded the highest (*P* ≤ 0.05) hemoglobin concentration in those fed 6 mg kg^−1^ compared to 3 mg kg^−1^. But no significant difference was noticed between fish-fed Nano-Cu at both levels of supplementation. Hematocrit value considerably improved (*P* ≤ 0.05) by supplementation of Cu, whereas the highest significant value demonstrated in fish provided 6 mg/kg^−1^ Nano-Cu. RBCs count and MCV levels rose markedly (*P* ≤ 0.05) in fish given Cu-supplemented diets either from Bulk or Nano source compared to those receiving the control diet, but RBCs count increased (*P* ≤ 0.05) as the inclusion level of Cu increased to 6 mg kg^−1^ from Bulk or Nano source. No significant variations were noticed in the values of MCH and MCHC among different groups. Moreover, WBCs, LYM, and GRAN cell count significantly improved (*P* ≤ 0.05) in fish administered 6 mg kg^−1^ Nano-Cu supplemented diets compared to those fed other diets.

**Table 4:** Hematological parameters of Nile tilapia as affected by dietary additives of bulk and Nano Copper

	Experimental treatments					± MSE	*P* value
	Control	Bulk-Cu (mg kg^−1^ diet)		Nano-Cu (mg kg^−1^ diet)			
		3	6	3	6		
Hb (g dl^−1^)	10.11^c^	11.50^b^	12.70^a^	12.55^a^	13.25^a^	0.578	0.0318
Hct (%)	28.27^d^	36.37^c^	38.33^b^	36.44^c^	40.10^a^	2.919	0.0001
RBCs (×10^12^ l^−1^)	2.57^c^	2.98^b^	3.14^a^	2.9^b^	3.23^a^	0.381	0.0001
MCV (fl)	110.09^b^	122.76^a^	122.82^a^	126.80^a^	124.25^a^	2.064	0.0032
MCH (pg)	39.44	39.63	40.96	44.08	41.66	1.196	0.4705
MCHC (g dl^−1^)	35.82	32.08	33.29	34.64	33.49	1.719	0.4531
WBCs (× 10^9^ l^−1^)	219.48^b^	170.27^d^	205.85^c^	216.88^b^	240.48^a^	2.562	0.0001
LYM (× 10^9^ l^−1^)	208.51^b^	161.76^d^	195.56^c^	206.04^b^	228.46^a^	2.434	0.0001
MID (× 10^9^ l^−1^)	10.20^b^	7.92^d^	9.57^c^	10.09^b^	11.18^a^	0.119	0.0001
GRAN (× 10^9^ l^−1^)	0.77^b^	0.60^d^	0.72^c^	0.76^b^	0.84^a^	0.059	0.0001

Means followed by different small letters in the same row are significantly different (*P* < 0.05, one-way ANOVA)

Abbreviations: *Hb* hemoglobin, *Hct* hematocrit, *RBCs* red blood cells count, *MCV* mean corpuscular volume, *MCH* mean corpuscular hemoglobin, *MCHC* mean corpuscular hemoglobin concentration, *WBCs* white blood cells (as a total count), *LYM* lymphocyte absolute count, *MID* mid-range absolute count, *GRAN* means granulocyte absolute count

### Plasma Metabolites

Plasma metabolites data are demonstrated in Table 5. Plasma ALT, AST, TC, and LDL-C levels were noticeably reduced (*P* ≤ 0.05) in fish given 3 and 6 mg kg^−1^ Cu-supplemented diets either Bulk or Nano, compared to those receiving the control diet. A fish-fed diet containing 3 mg kg^−1^ Nano-Cu revealed the best (*P* ≤ 0.05) values of plasma albumin, total protein, and globulins. Plasma HDL-C highest concentrations (*P* ≤ 0.05) were reported in fish fed diet supplemented with 6 mg/kg^−1^ either Bulk or Nano Cu. Plasma TG and VLDL-C values reduced considerably in a fish-fed diet supplemented with Cu compared to those administered the control diet, whereas values declined as Cu supplementation level increased either from Bulk or Nano source.

**Table 5:** Alanine aminotransferase, aspartate aminotransferase, plasma total protein, albumin, globulin, albumin/globulin ratio, and plasma lipids profiles of Nile tilapia as affected by dietary additives of bulk and nano copper

	Experimental treatments					± MSE	*P* value
	Control	Bulk-Cu (mg kg^−1^ diet)		Nano-Cu (mg kg^−1^ diet)			
		3	6	3	6		
ALT (U l^−1^)	34.78^a^	22.36^c^	23.45^b^	22.08^c^	21.60^c^	0.294	0.0001
AST (U l^−1^)	93.53^a^	77.68^b^	76.27^b^	75.42^b^	75.12^b^	0.812	0.0001
TP (g dl^−1^)	2.57^c^	2.54^c^	2.71^b^	3.26^a^	2.85^b^	0.203	0.0140
Al (g dl^−1^)	0.90^c^	0.92^c^	0.98^bc^	1.15^a^	1.04^ab^	0.034	0.0052
Gl (g dl^−1^)	1.67^c^	1.62^c^	1.67^c^	2.11^a^	1.87^b^	0.197	0.0137
Al/Gl ratio	0.57^b^	0.58^b^	0.65^a^	0.54^b^	0.54^b^	0.044	0.8014
TC (mg dl^−1^)	179.53^a^	158.10^b^	157.86^b^	154.10^b^	158.42^b^	2.722	0.0011
TG (mg dl^−1^)	180.14^a^	138.27^b^	125.18^c^	135.20^b^	132.63^bc^	2.548	0.0001
HDL-C (mg dl^−1^)	35.43^c^	44.91^b^	46.01^a^	44.26^b^	46.53^a^	0.028	0.0001
LDL-C (mg dl^−1^)	108.08^a^	85.53^b^	86.81^b^	82.80^b^	85.37^b^	2.940	0.0016
VLDL-C (mg dl^−1^)	36.03^a^	27.65^b^	25.04^c^	27.04^b^	26.53^bc^	0.509	0.0001

Means followed by different small letters in the same row are significantly different (*P* < 0.05, one-way ANOVA)

Abbreviations: *ALT* alanine aminotransferase, *AST* aspartate aminotransferase, *TP* total protein, *Al* albumin, *Gl* globulin, *Al/Gl* ratio albumin/globulin ratio, *TC* total cholesterol, *TG* triglycerides, *HDL-C* high-density lipoprotein cholesterol, *LDL-C* low-density lipoprotein cholesterol, *VLDL-C* very low-density lipoprotein cholesterol

### Plasma Immune and Antioxidant Biomarkers

Plasma immune and antioxidant biomarkers are found in Table 6. Plasma lysozyme activity rose (*P* ≤ 0.05) in fish that received dietary Cu supplementation, whereas the highest values were reported in fish that received Nano-Cu compared to fish fed Bulk-Cu and the control diets. Plasma CAT, GPX, and SOD levels improved (*P* ≤ 0.05) as dietary Cu supplementation was raised in comparison to the levels reported in the control group, whereas the best (*P* ≤ 0.05) values of CAT and GPX were seen in fish given diet supplemented with 6 Nano-Cu mg/kg^−1^. While plasma SOD showed the highest (*P* ≤ 0.05) level in a fish-fed diet supplemented with 6 mg/kg^−1^ either Bulk or Nano-Cu. Dietary Cu supplementation markedly lowered (*P* ≤ 0.05) the plasma MDA levels compared to the plasma MDA level of fish fed Cu free diet (control), whereas the lowest value was recorded in fish fed 6 mg/kg Nano-Cu supplemented diet.

**Table 6:** Lysozyme, catalase, superoxide dismutase, glutathione peroxidase, glutathione, glutathione S-transferases, glutathione-disulfide reductase, and malondialdehyde of Nile tilapia as affected by dietary additives of bulk and Nano Copper

	Experimental treatments					± MSE	*P* value
	Control	Bulk-Cu (mg kg^−1^ diet)		Nano-Cu (mg kg^−1^ diet)			
		0.3	0.6	0.3	0.6		
LZM (ng ml^−1^)	0.70^c^	0.80^b^	0.81^b^	0.84^a^	0.85^a^	0.055	0.0001
CAT (ng ml^−1^)	50.50^d^	51.10^d^	58.30^b^	52.80^c^	63.43^a^	1.389	0.0001
SOD (U ml^−1^)	25.06^c^	29.15^b^	34.26^a^	28.23^b^	33.86^a^	0.395	0.0001
GPx (ng ml^−1^)	25.94^d^	27.94^c^	31.08^b^	26.10^d^	32.42^a^	0.156	0.0001
MDA (nmol ml^−1^)	73.95^a^	69.20^b^	69.17^b^	66.64^b^	62.74^c^	0.806	0.0001

Means followed by different small letters in the same row are significantly different (*P* < 0.05, one-way ANOVA)

Abbreviations: *LZM* lysozyme, *CAT* catalase, *SOD* superoxide dismutase, *GPx* glutathione peroxidase, *GSH* glutathione, *GST* glutathione S-transferases, *GSR* glutathione-disulfide reductase, *MDA* malondialdehyde

### Fish Fillet Proximate Analysis

The proximate analysis of fish fillet nutrients is presented in Table 7. Fillets of fish-fed Nano-Cu-supplemented diets showed a marked decline (*P* ≤ 0.05) in moisture and fat contents, while crude protein, ash, and Cu contents considerably increased in the fillet by dietary supplementation of Nano-Cu at both levels 3 and 6 mg kg^−1^.

**Table 7:** Proximate analysis of flesh of Nile tilapia as affected by dietary additives of bulk-Se and Nano copper

	Experimental treatments					± MSE	*P* value
	Control	Bulk-Cu (mg kg^−1^ diet)		Nano-Cu (mg kg^−1^ diet)			
		3	6	3	6		
Chemical composition of fish flesh							
Moisture (%)	74.80^a^	74.61^ab^	74.10^bc^	73.72^c^	73.67^c^	0.185	0.0076
Protein (%)	18.79^d^	19.41^c^	19.89^b^	20.18^a^	20.37^a^	0.048	0.0001
Fat (%)	2.93^a^	2.50^b^	2.31^c^	2.20^ cd^	2.05^d^	0.015	0.0001
Ash (%)	2.01^e^	2.25^d^	2.43^c^	2.59^b^	2.73^a^	0.032	0.0001
Cu (mg kg^−1^)	0.49^d^	0.51^bc^	0.63^b^	1.18^a^	1.23^a^	0.014	0.0001

Means followed by different small letters in the same row are significantly different (*P* < 0.05, one-way ANOVA)

Abbreviations: *Cu* copper

## Discussion

### Growth Indices

In the present study, fish growth traits, feed utilization, and body indices revealed a significant improvement (*P* ≤ 0.05) in tilapia by dietary Cu supplementation, whereas fish received diets containing 3 and 6 Nano-Cu mg kg^−1^ demonstrated the best performance compared to the performance of those fed diets supplemented with 3 and 6 Bulk-Cu mg kg^−1^ and the control diet. Typically, when the dietary copper increases from zero to 3 and 5 mg kg^−1^, it stimulates the growth of grouper [7] and tilapia [10], respectively. Copper is a vital trace element that is needed to improve feed quality for enhancing fish growth.

The best performance noticed in fish-provided diets supplemented with Nano-Cu. These findings illustrated that Nano-Cu additive can improve fish growth, survival, and feed efficiency at both levels of supplementation. The role of Nano-Cu compounds as a dietary source of Cu to enhance and promote fish performance has been confirmed in some fish species. [43] suggested that Nano Cu can enhance growth efficiently more than inorganic Cu (CuSO_4_) for satisfying the dietary Cu requirement of Russian sturgeon (*Acipenser gueldenstaedtii*) and can lower the amount of Cu additive. It indicates that the Nano-Cu utilization rate is 1.5–2 higher than in the CuSO_4_ for the dietary Cu requirements of Russian sturgeon. A similar finding in red sea bream also assured the growth-promoting action of Nano-Cu particles [23]. This growth-promoting effect of Nano-Cu is explained by [43], which illustrates that Nano-Cu improves the Cu absorption and deposition efficiency if the Cu level by the fish species in the diet is at the normal required level. The higher availability and absorption efficiency of Nano-Cu source herein are due to the small size and diameter of nanoparticles. The small diameter of copper particles facilitates its uptake as an intact nanoparticle [44]. Growth and feed utilization stimulating effect of Nile tilapia diets supplemented with Nano mineral elements such as Se and Zn has been also observed by [30, 33]. The inorganic salts of Cu have a less growth-promoting effect and lower availability than the Nano-Cu; it may be due to during the absorption, Cu ions may convert to indigestible compounds with inhibitors compounds (tricalcium phosphate, phytic acid, and fiber), which in turn hinder Cu absorption [45]. The lowest performance recorded in fish fed the control diets may be due to the Cu present in the control diet only from the plant ingredients. The presence of phytic acid, which binds to divalent and trivalent cations including Fe, Zn, Mg, Cu, Mn, and Ca and may reduce these nutrients’ bioavailability [46, 47], is typically associated with oilseeds and cereal grains.

### *Intestinal Morphometry*

Our observed findings from intestinal morphometry examination revealed the antioxidant effect of the Cu, as it protects against cellular oxidative damage as reported previously by [46]. This explained the improvement of the intestinal morphology in fish fed Cu-supplemented diets, compared to those fed the control diet. The significantly highest values of villi height, width, goblet cell numbers, AAV, and MSR measured in fish fed Nano-Cu compared to those fed Bulk indicate the active absorption status of the intestinal villi because of the higher biological effects, antioxidant activities, and the better bioavailability of Cu from Nanoparticles [29]. The Cu ions from Bulk sources may combine with the insoluble compounds, such as phytic acid which is present in plant ingredients (corn and soybean meal) in the diet, lowering its availability and its biological effects.

### Blood Assay

Compared to the hemoglobin concentration of fish fed the control diet, hemoglobin concentration improved (*P* ≤ 0.05) by dietary supplementation of Cu either from Bulk or Nano-Cu, whereas fish fed Bulk-Cu recorded the highest (*P* ≤ 0.05) hemoglobin concentration in those fed 6 mg/kg^−1^ compared to 3 mg/kg^−1^. But no significant difference was noticed between fish-fed Nano-Cu at both levels of supplementation 3 and 6 mg/kg^−1^. Hematocrit value considerably improved (*P* ≤ 0.05) by supplementation of Cu, whereas the highest significant value was demonstrated in fish fed 6 mg/kg^1^ Nano-Cu. RBCs count and MCV levels increased markedly (*P* ≤ 0.05) in fish fed Cu supplemented diets either from Bulk or Nano source compared to those fed the control diet, but RBCs count increased (*P* ≤ 0.05) as the supplementation level of Cu increased to 6 mg/kg^−1^ from Bulk or Nano source. These results support the idea of the biological function of Cu in the synthesis of hemoglobin and metabolism of iron as Cu is a component of the ceruloplasmin enzyme which undergoes the ferroxidase activity needed for iron transportation into the blood circulation [3], and it works as an antioxidant in plasma [47], which is important to maintain RBCs and WBCs viability and prevent its oxidative damage. Also, Cu plays a role in collagen production needed for RBC formation. It stimulates the lysyl oxidase enzyme that is required for collagen maturation [3].

The lowest values of WBCs count in the control diet (Cu-free) may base on that Cu deficiency suppresses certain white cell populations such as macrophages in mammal and neutropenia in humans [48].

The highest value of hemoglobin recorded in fish-fed Nano-Cu at low and high levels of supplementation 3 and 6 mg kg^−1^, while the high level recorded only in fish-fed 6 mg kg^−1^ Bulk-Cu could be due to the better bioavailability and utilization of Cu from nanoparticles. The high bioavailability also explained the high values of hematocrit, white blood cell (WBCs), lymphocyte (LYM), Mid-Sized Cells (MID), and granulocyte (GRAN) in fish-fed Nano-Cu compared to those fed other diets.

### *Plasma Immune and Antioxidants Biomarkers*

Plasma lysozyme activity increased (*P* ≤ 0.05) in fish by dietary Cu supplementation, whereas the highest values were reported in fish-fed Nano-Cu compared to fish-fed Bulk-Cu and the control diets. The obtained data herein proved the beneficial role of Cu in enhancing the immune response of tilapia. Cu performs several roles in the immune system, and the alteration in non-specific immunity is linked to the dietary copper concentration. Copper deficiency reduces mammalian T cell and macrophage proliferation, cytokine production, and antibody production and increases the disease incidence [49]. The highest activity of lysozyme was measured in Nano-Cu groups, agreed with the data obtained by [43], which observed the higher lysozyme, C3, and IgM contents in sturgeon fed the diet with Cu content at 4.29 mg kg^−1^ in CuO Nano with the least cumulative mortality, suggesting the high absorption and better utilization of Cu from Nano supplement. The relationship between dietary copper and innate immunity in shrimp, such as *Penaeus monodon* and *Penaeus vannamei*, has been illustrated [17].

Plasma CAT, GPX, and SOD levels improved (*P* ≤ 0.05) as dietary Cu supplementation increased compared to the levels reported in the control group, whereas the best (*P* ≤ 0.05) values of CAT and GPX were found in fish fed diet supplemented with 6 Nano-Cu mg/kg^−1^, while plasma SOD showed the highest (*P* ≤ 0.05) level in a fish-fed diet supplemented with 6 mg/kg^−1^ either Bulk or Nano-Cu. Dietary Cu supplementation markedly lowered (*P* ≤ 0.05) the plasma MDA levels compared to the plasma MDA level of fish fed Cu free diet (control), whereas the lowest value was recorded in fish fed 6 mg/kg Nano-Cu supplemented diet. The obtained data could be attributed to the potential role of Cu in raising the activity of plasma antioxidant enzymes. [43] recorded low levels of Cu–Zn SOD, T-AOC, and ceruloplasmin activity, but the MDA content was high in sturgeon fed the control diet (Cu-free). Also, they noticed that the antioxidation of fish-fed diets with 4.29 mg Cu/kg in CuO-Nano was equal to that of fish fed 6.34 mg Cu/kg in CuSO_4_, indicating that CuO-Nano has a better ability to enhance antioxidant capacity in Russian sturgeon than CuSO_4_ when the dietary Cu content in diet is at the required level by sturgeon. As reported by [2] Cu is a component of ceruloplasmin that has an antioxidant effect in plasma, protects cells against oxidative damage [46], copper, iron, and selenium, controls the activity of superoxide dismutase, catalase, and glutathione peroxidase involved in the defense mechanisms against reactive oxygen species, as scavenging free radicals [3, 11].

### Plasma Metabolites

Plasma ALT, AST, TC, and LDL-C levels were noticeably reduced (*P* ≤ 0.05) in fish fed 3 and 6 mg/kg^−1^ either Bulk or Nano Cu supplemented diets compared to those fed the control diet. Our finding can be explained by, the antioxidative effects of Cu and the protection of hepatic cells against oxidative damage, which infer the significantly lower levels of hepatic enzymes in fish-fed Cu-supplemented diet versus those fed the control (Cu-free). [2, 46] reported that Cu is a cofactor superoxide dismutase and ceruloplasmin, which have a potent antioxidant effect as a free radical scavenger and protect the cell from the oxidative damage. The results revealed that Cu has a cholesterol-lowering effect the same as reported by [50] who noticed that copper deficiency in animals and humans causes glucose intolerance and hypercholesterolemia; one mechanism for this being increased levels of hydroxymethylglutaryl-coenzyme A (HMG-CoA) reductase, a key enzyme in the cholesterol synthesis pathway.

Fish-fed diet supplemented with 3 mg kg^−1^ Nano-Cu recorded the highest (*P* ≤ 0.05) values of plasma albumin, total protein, and globulins. Plasma HDL-C values revealed a significant increase by dietary Cu supplementation compared to the control group, whereas the increase of the concentration (*P* ≤ 0.05) as dietary Cu supplementation increased to 6 mg kg^−1^ either Bulk or Nano Cu. This agreed with [51] who observed that copper deficiency increases the susceptibility of high-density lipoprotein (HDL) to oxidation, indicating that Cu protects lipoprotein from oxidation. Our results indicate the role of Cu as a cofactor for some enzymes involved in cell metabolism, protein synthesis, and immunoglobulin production [3, 49]. Plasma TG and VLDL-C values reduced considerably in the fish-fed diet supplemented with Cu compared to those fed the control diet, whereas values decreased as the Cu supplementation level increased to 6 mg kg^−1^ either from Bulk or Nano source. This indicates that Cu has a lipid-lowering effect. Likewise, in many studies in rodents and other animal models, it has been proposed that copper deficiency is a factor in the development of Fatty-Liver Disease, can alter lipid metabolism, and is a significant factor in diseases associated with dyslipidemia [52]. Further, [53] showed that copper supplementation significantly reduced levels of oxidized serum LDL in a middle-aged adult population. In copper deficient Rat triglycerides, phospholipids, and cholesterol in LDL and HDL increased twofold or more compared to control; the VLDL composition of copper-deficient animals changed most significantly with a sixfold increase in triglycerides.

Also, the increased levels of total protein herein indicate a healthy liver, as a result of the antioxidant effect of the Cu. Total plasma protein is the level of albumin and globulin in the blood. Albumin is the major component of blood protein and is responsible for nutrient transportation and the maintenance of osmotic balance, and globulin is addressed in the defense mechanism of animals [54].

### Fish Flesh Proximate Analysis

In the present data, the flesh of fish showed a significant reduction in moisture and fat contents. While crude protein, ash, and Cu contents considerably increased in the flesh by dietary supplementation of Nano-Cu at both levels 3 and 6 mg/kg^−1^ compared to the flesh of fish-fed Bulk-Cu and the control diets. These results suggest the higher bioavailability, absorption, and efficient utilization of Cu ions from Nano-Cu particles compared to the inorganic Cu. As a result, the Cu deposition rate increased in the flesh and improved ash and protein retention efficiency, indicating the efficient utilization of protein for supporting growth. [43] found that the improvement in the Cu apparent digestibility of CuO Nano effectively rises their retention rate and growth effect in Russian sturgeon compared to fish fed the same amount of CuSo_4_. It has been established previously by [55] the role of Cu as an essential element required for growth, hemoglobin production, and protein syntheses such as collagen, also as a cofactor for Cu-dependent enzymes and a component of specific proteins, which stimulate important metabolic processes.

## Conclusion

Due to copper’s high retention in fish bodies and consequent decrease in its concentration in the environment to produce an eco-friendly environment, the current findings of the study highlight and confirm the significance of using copper nanoparticle form in aqua-feeds. To further improve Nile tilapia performance, activate the digestive enzymes, and increase antioxidant enzyme capacity, copper inclusion in Nano-Cu form is a helpful application. The current findings of the study emphasize and validate the relevance of using copper nanoparticle in aqua-feeds due to copper's high retention in fish bodies and resulting decrease in its concentration in the environment. Copper inclusion in Nano-Cu form is a useful application to enhance Nile tilapia performance, activate the digestive enzymes, and raise antioxidant enzyme capacity.

## Data Availability

Data of the present article are not available.

## References

[1] World Health Organization & Food and Agriculture Organization of the United Nations (2020) The Joint FAO/WHO Expert Meetings on Nutrition ( JEMNU) : nitrogen to protein conversion factors for soy-based and milkbased ingredients used in infant formula and follow-up formula: report of the meeting of the expert panel: Geneva, Switzerland, 16–17 July 2019

[2] Watanabe T, Kiron V, Satoh S (1997). Trace minerals in fish nutrition. Aquaculture.

[3] Lall S (2002). The minerals.

[4] Kamunde C, Clayton C, Wood CM (2002). Waterborne vs. dietary copper uptake in rainbow trout and the effects of previous waterborne copper exposure. Am J Physiol Regul Integr Comp Physiol.

[5] Mondal M (2007). Influence of dietary inorganic and organic copper salt and level of soybean oil on plasma lipids, metabolites and mineral balance of broiler chickens. Anim Feed Sci Technol.

[6] Berntssen MH, Lundebye A-K, Maage A (1999). Effects of elevated dietary copper concentrations on growth, feed utilisation and nutritional status of Atlantic salmon (Salmo salar L.) fry. Aquaculture.

[7] Richards MP (1997). Trace mineral metabolism in the avian embryo. Poult sci.

[8] Gatlin DM, Wilson RP (1986). Dietary copper requirement of fingerling channel catfish. Aquaculture.

[9] Julshamn K (1988). Effect of dietary copper on the hepatic concentration and subcellular distribution of copper and zinc in the rainbow trout *(Salmo gairdneri)*. Aquaculture.

[10] Shiau S, Ning Y (2003). Estimation of dietary copper requirements of juvenile tilapia, Oreochromis niloticus × O. aureus. Anim Sci.

[11] Valko M (2007). Free radicals and antioxidants in normal physiological functions and human disease. Int J biochem cell biol.

[12] Fedeli D, Carloni M, Falcioni G (2010). Oxidative damage in trout erythrocyte in response to “in vitro” copper exposure. Mar environ res.

[13] Ogino G, Yang GY (1980). Requirements of carp and rainbow trout for dietary manganese and copper. Bull Jpn Soc Sci Fish.

[14] NRC (2011) Nutrient requirements of fish and shrimp. The National Academies Press, Washington DC

[15] Knox D, Cowey CB, Adron JW (1982). Effects of dietary copper and copper: zinc ratio on rainbow *trout Salmo gairdneri*. Aquaculture.

[16] Murai T, Andrews JW, Smith RG (1981). Effects of dietary copper on channel catfish. Aquaculture.

[17] Lee M-H, Shiau S-Y (2002). Dietary copper requirement of juvenile grass shrimp, *Penaeus monodon,* and effects on non-specific immune responses. Fish Shellfish Immunol.

[18] Tan XY (2011). Dietary copper requirement of juvenile yellow catfish *Pelteobagrus fulvidraco*. Aquac nutr.

[19] Tang Q (2013). Effects of dietary copper on growth, digestive, and brush border enzyme activities and antioxidant defense of hepatopancreas and intestine for young grass carp *(Ctenopharyngodon idella)*. Biol Trace Elem Res.

[20] Shao X-p (2010). Effects of dietary copper sources and levels on performance, copper status, plasma antioxidant activities and relative copper bioavailability in *Carassius auratus gibelio*. Aquaculture.

[21] Cao J (2014). Dietary copper requirements of juvenile large yellow croaker *Larimichthys croceus*. Aquaculture.

[22] White JE, Catallo WJ, Legendre BL (2011). Biomass pyrolysis kinetics: a comparative critical review with relevant agricultural residue case studies. J Anal Appl Pyrolysis.

[23] El Basuini MF (2016). Effect of different levels of dietary copper nanoparticles and copper sulfate on growth performance, blood biochemical profiles, antioxidant status and immune response of red sea bream *(Pagrus major)*. Aquaculture.

[24] Wang W (2009). Effects of dietary copper on survival, growth and immune response of juvenile abalone. Haliotis Discus Hannai Ino Aquaculture.

[25] Clearwater SJ, Farag AM, Meyer J (2002). Bioavailability and toxicity of dietborne copper and zinc to fish. Comp Biochem Physiol Part - C: Toxicol Pharmacol.

[26] Spears J, Kegley E, Mullis L (2004). Bioavailability of copper from tribasic copper chloride and copper sulfate in growing cattle. Anim Feed Sci Technol.

[27] Apines-Amar MJS (2004). Amino acid-chelate: a better source of Zn, Mn and Cu for rainbow trout. Oncorhynchus Mykiss Aquaculture.

[28] Paripatananont T, Lovell RT (1995). Chelated zinc reduces the dietary zinc requirement of channel catfish. Ictalurus Punctatus Aquaculture.

[29] Bellmann S (2015). Mammalian gastrointestinal tract parameters modulating the integrity, surface properties, and absorption of food-relevant nanomaterials. Wiley Interdisc Reviews: Nanomed Nanobiotechnol.

[30] Ibrahim MS (2021). Nanoselenium versus bulk selenium as a dietary supplement: effects on growth, feed efficiency, intestinal histology, haemato-biochemical and oxidative stress biomarkers in Nile tilapia *(Oreochromis niloticus* Linnaeus, 1758*)* fingerlings. Aquac Res.

[31] Caruso M, Demonte A, Neves VA (2012). Histomorphometric study of role of lactoferrin in atrophy of the intestinal mucosa of rats. Health.

[32] Reitman S, Frankel S (1957). A colorimetric method for the determination of serum glutamic oxalacetic and glutamic pyruvic transaminases. Am J Clin Pathol.

[33] Ibrahim MS (2022). Nano zinc versus bulk zinc form as dietary supplied: effects on growth, intestinal enzymes and topography, and hemato-biochemical and oxidative stress biomarker in Nile tilapia (*Oreochromis niloticus* Linnaeus, 1758). Biol Trace Elem Res.

[34] Lewis S, Bain B, Bates ID (2001) Lewis practical hematology. 9 [sup] th ed. Churchill Livingstone, London

[35] Sampathy K, James R, Ali KMA (1998). Effects of copper and zinc on blood parameters and prediction of their recovery in *Oreochromis mossambicus* (pisces: cichlidae). Indian J Fish.

[36] Coles EH (1974) Plasma proteins. In: Veterinary clinical pathology, 2nd ed. WB Saunders Co., Philadelphia, pp 558–560

[37] Henry RJ (1964) Colorimetric determination of total protein. In: Clinical Chemistry. Harper and Row Publ, New York

[38] Friedewald WT, Levy RI, Fredrickson DS (1972). Estimation of the concentration of low-density lipoprotein cholesterol in plasma, without use of the preparative ultracentrifuge. Clin chem.

[39] Schäperclaus W, Kulow H, Schreckebach K (1992) Infectious abdominal dropsy. In: Schäperclaus W (ed.) fish disease, vol 1. Akademie- Verlag, Berlin, pp 401–458

[40] Bancroft J, Stevens A, Turner R (1982) Theory and practice of histological techniques (2nd ed.). Churchill Living Stone, New York

[41] Mohammady EY (2021). Comparative effects of dietary zinc forms on performance, immunity, and oxidative stress-related gene expression in Nile tilapia. Oreochromis Niloticus Aquaculture.

[42] Lee MH (1995) Official methods of analysis of AOAC International (16th edn). AOAC International, Rockville

[43] Wang H (2018). Comparison of copper bioavailability in copper-methionine, nano-copper oxide and copper sulfate additives in the diet of Russian sturgeon *Acipenser gueldenstaedtii*. Aquaculture.

[44] Florence A (1995). Factors affecting the oral uptake and translocation of polystyrene nanoparticles: histological and analytical evidence. J Drug target.

[45] Kim B-E, Nevitt T, Thiele DJ (2008). Mechanisms for copper acquisition, distribution and regulation. Nat Chem Biol.

[46] Sevcikova M (2011). Metals as a cause of oxidative stress in fish: a review. Vet Med.

[47] Luza SC, Speisky HC (1996). Liver copper storage and transport during development: implications for cytotoxicity. Am J Clin Nutr.

[48] Babu U, Failla ML (1990). Respiratory burst and candidacidal activity of peritoneal macrophages are impaired in copper-deficient rats. J Nutr.

[49] Dorton KL (2003). Effects of copper source and concentration on copper status and immune function in growing and finishing steers. Anim Feed Sci Technol.

[50] Klevay LM (1987). Hypertension in rats due to copper deficiency. Nutr Rep Int.

[51] Rayssiguier Y (1993). Copper deficiency increases the susceptibility of lipoproteins and tissues to peroxidation in rats. J Nutr.

[52] Aigner E (2010). A role for low hepatic copper concentrations in nonalcoholic fatty liver disease. Am J Gastroenterol ACG.

[53] DiSilvestro RA (2012). A randomized trial of copper supplementation effects on blood copper enzyme activities and parameters related to cardiovascular health. Metabolism.

[54] Thomas J, Feldman BF, Zinkl JG, Jain NC (2000). Overview of plasma proteins. Schalm’s veterinary hematology.

[55] O'Dell BL (1976). Biochemistry of copper. Med Clin North Am.

